# A biphasic oxidation of alcohols to aldehydes and ketones using a simplified packed-bed microreactor

**DOI:** 10.3762/bjoc.5.17

**Published:** 2009-04-29

**Authors:** Andrew Bogdan, D Tyler McQuade

**Affiliations:** 1 Department of Chemistry and Chemical Biology, Cornell University, Ithaca, NY, 14853 USA; 2 Department of Chemistry and Biochemistry, Florida State University, Tallahassee, FL 32306, USA. Fax (850) 644-8281

**Keywords:** alcohol oxidation, flow chemistry, heterogeneous catalysis, microreactors, TEMPO

## Abstract

We demonstrate the preparation and characterization of a simplified packed-bed microreactor using an immobilized TEMPO catalyst shown to oxidize primary and secondary alcohols via the biphasic Anelli-Montanari protocol. Oxidations occurred in high yields with great stability over time. We observed that plugs of aqueous oxidant and organic alcohol entered the reactor as plugs but merged into an emulsion on the packed-bed. The emulsion coalesced into larger plugs upon exiting the reactor, leaving the organic product separate from the aqueous by-products. Furthermore, the microreactor oxidized a wide range of alcohols and remained active in excess of 100 trials without showing any loss of catalytic activity.

## Introduction

Microreactors have gained attention because they can help run chemical transformations more efficiently, more selectively, and with a higher degree of safety [1–12]. Alcohol oxidations are well suited for microreactors due to high by-product formation, catalyst contamination and safety concerns often associated with scale-up in batch reactors [13]. Recent developments in microreactor technology and solid-supported catalysis have aimed to solve these issues. Numerous flow oxidations have been presented in the literature [13–16], however many rely on stoichiometric reagents [17–18], suffer from catalyst deactivation [19–20], or rely on soluble catalysts [21], all of which limit their potential as continuous flow processes. Realizing this, we developed a catalytic packed-bed microreactor that could be used for the continuous flow oxidation of alcohols to aldehydes or ketones.

Oxidations that do not require transition metal catalysts are particularly appealing since there is neither leaching nor the need for catalyst regeneration [22]. Nitroxyl radicals, such as 2,2,6,6-tetramethylpiperidine-1-oxyl (TEMPO), immobilized on silicates [23–32], fluorous supports [33], soluble and insoluble polymers [22,34–36], magnetic nanoparticles [37], and ionic liquids [38] have been extensively studied and are useful because the oxidation conditions are mild, selective, and the catalysts do not require regeneration. TEMPO, however, is expensive and difficult to remove from reaction mixtures. For these reasons, TEMPO immobilization is an important goal because it enables facile removal and recycling.

We recently reported the development of an effective solid support for use in packed-bed microreactors [39], AMBERZYME^®^ Oxirane (AO, **1**), a commercially available resin with pendant epoxide functionalities designed for enzyme immobilization. AO is readily functionalized with a range of catalysts and works well as packing material for flow chemistry [39]. In this report, we demonstrate the immobilization of TEMPO and its use in a flow system using the Anelli-Montanari protocol for the oxidation of primary and secondary alcohols [30,40]. Our simplified reactor is advantageous because the reactions not only run continuously, but since the microreactor is made of cheap, disposable fluoroelastomeric tubing, wall oxidation that is commonplace with metal microchannels is not observed [21]. The narrow dimensions of the microreactor also allow excellent heat transfer, increasing the safety of large-scale oxidations. Furthermore, it was determined that the use of a packed-bed microreactor facilitates efficient mixing of a biphasic system without destroying the solid support, a common problem with stirred systems [4].

## Results and Discussion

**Scheme 1:**
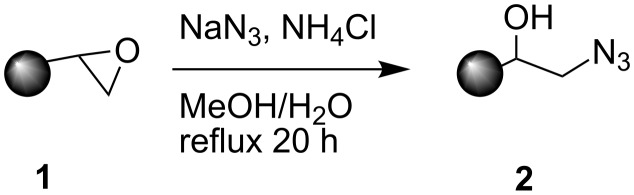
Preparation of azide-modified AO resin **2**.

**Scheme 2:**
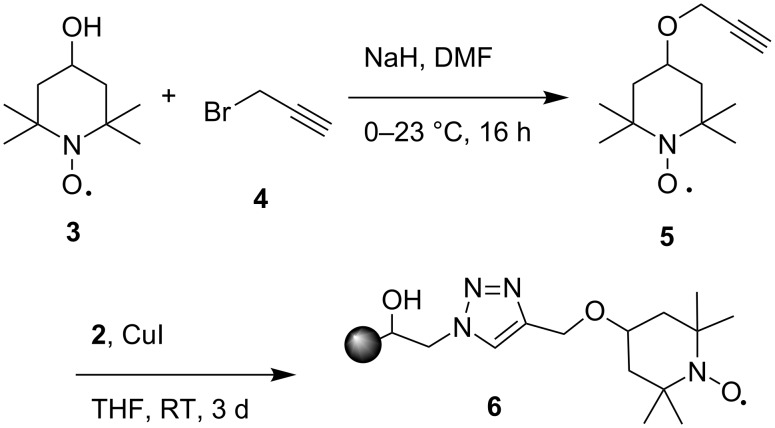
Preparation of AO-TEMPO **6**.

Previously we reported that catalysts functionalized with an acetylene moiety can be tethered to AO using a Huisgen cycloaddition [39]. Azide-functionalized AO resin (AO-N_3_, **2**) was prepared by treating AO with sodium azide (Scheme 1) [41]. The coupling of 4-hydroxy-TEMPO **3** with propargyl bromide (**4**) using sodium hydride yielded the acetylene-modified TEMPO species **5** [33–34,37]. TEMPO derivative **5** was then covalently bound to AO-N_3_ using copper(I) iodide [42–45], yielding the TEMPO-functionalized resin (AO-TEMPO, **6**, Scheme 2, 0.46 mmol TEMPO/g resin).

**Figure 1:**
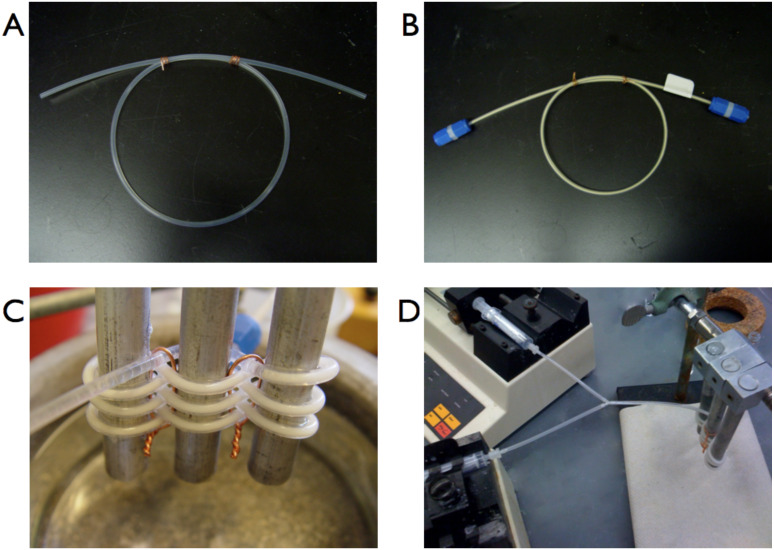
The simplified microreactor setup. Empty tubing (A) is packed with functionalized AO resin and attached to caps (B). The packed bed is woven between metal bars (C) and connected to syringe pumps (D).

**Figure 2:**
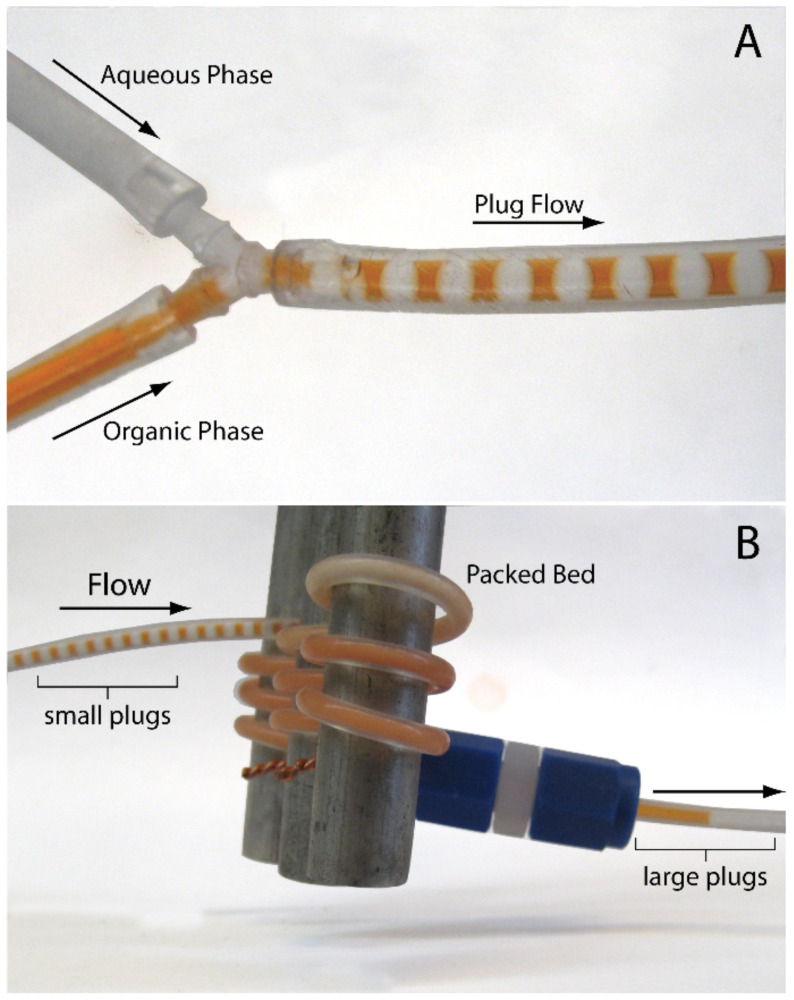
The organic (colored solution) and aqueous phases (colorless solution) forming plugs at the Y-junction (A). The phases mix upon reaching the packed bed, leading to a coalescence of drops at the outlet of the microchannel (B).

Using a simplified procedure developed by ourselves and others [39,46–49], flow reactions were performed by packing fluoroelastomeric tubing (60 cm, 1.6 mm i.d.) with the AO-TEMPO resin. The tubing was subsequently woven between metal bars, to improve mixing and to enable facile microreactor cooling (Figure 1). A Y-junction placed at the inlet of the microreactor allowed the immiscible bleach (adjusted to pH 9.1 using NaHCO_3_) and organic alcohol solutions to form plugs before reaching the packed bed (Figure 2A). When passing through the AO-TEMPO catalyst bed, the plugs emulsified, as indicated by visual inspection and by the plug coalescence at the microchannel outlet (Figure 2B). The effective mixing was later supported by the high yields and conversions that were achieved for this biphasic reaction.

**Scheme 3:**
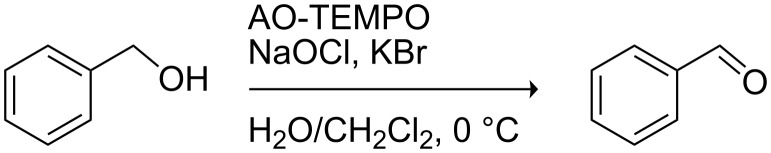
The AO-TEMPO-catalyzed oxidation of benzyl alcohol.

Preliminary reactions were performed using benzyl alcohol as the test substrate in order to establish the optimal flow conditions (Scheme 3). A solution of benzyl alcohol in CH_2_Cl_2_ (0.2 M), an aqueous NaOCl solution (0.25 M, adjusted to pH 9.1 using NaHCO_3_), and an aqueous KBr solution (0.5 M) were prepared. The organic phase was loaded into one syringe and a mixture of NaOCl and KBr (30 μL KBr solution per mL NaOCl solution) was added to another. The syringes were placed on separate syringe pumps and the flow rates were regulated such that 1.0 equiv alcohol min^−1^, 1.5 equiv NaOCl min^−1^, and 0.10 equiv KBr min^−1^ were delivered to the packed bed. Various flow rates were examined to determine the optimal flow conditions for the oxidation. For data relating flow rate to residence time, see Supporting Information File 1. During our optimization studies, it was shown that a total flow rate of 100 µL min^−1^ (aqueous flow rate 56 µL min^−1^ and organic flow rate 44 µL min^−1^, approximately 4.8 min residence time) afforded quantitative conversion of benzyl alcohol to benzaldehyde, indicating that efficient mixing was occurring in the column at this flow rate. Faster flow rates (200 or 400 μL min^−1^) could also be used to obtain higher outputs of benzaldehyde, however these reaction conditions did not provide complete conversion of starting material.

Using these optimized flow conditions for the benzyl alcohol oxidation, a number of different substrates were examined to test the generality of the AO-TEMPO packed-bed microreactor. High conversions were achieved when using both aromatic and aliphatic alcohols (Table 1, Entries 1–6). Secondary alcohols, which are known to be oxidized at a slower rate than primary alcohols, could effectively be oxidized to ketones by increasing the equivalents of NaOCl with respect to the alcohol concentration (Table 1, Entries 7–9). Primary alcohols were shown to be oxidized selectively over secondary alcohols (Table 1, Entry 10). Interestingly, it was also demonstrated that ethyl acetate was almost as effective a solvent as methylene chloride, opening the possibility of making this process “green” (Table 1, Entry 11). While reactions range from modest to high yields, systems that do not perform as efficiently could readily be optimized to afford higher conversions and yields. For the purposes of this paper however, we were solely testing the generality of the method and, therefore, did not optimize every substrate. Similar to our previous packed-bed systems, these AO-TEMPO microchannels showed a high degree of recyclability, in some cases being used in excess of 100 trials without any apparent loss of catalytic activity. Channels also maintained a high activity after three months of not being used.

**Table 1:** Oxidation of alcohols using AO-TEMPO packed-bed microreactor.

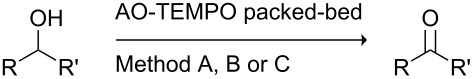						
Entry	Alcohol	Method	Solvent	Product	Conversion^d^	Yield^d^

1	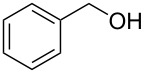	A^a^	CH_2_Cl_2_	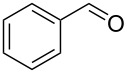	>99%	95%
2	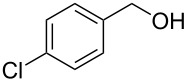	A	CH_2_Cl_2_	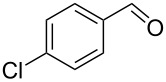	>99%	93% (86%)^e^
3	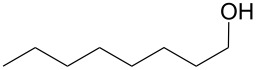	A	CH_2_Cl_2_	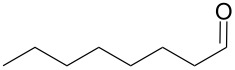	88%	85%
4	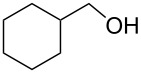	A	CH_2_Cl_2_	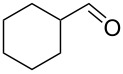	89%	86%
5	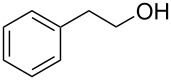	A	CH_2_Cl_2_	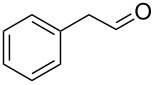	88%	71%
6	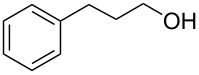	A	CH_2_Cl_2_	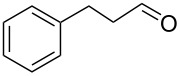	80%	74%
7	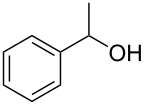	B^b^	CH_2_Cl_2_	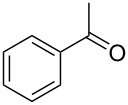	>99%	95%
8	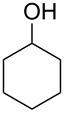	B	CH_2_Cl_2_	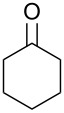	95%	84%
9	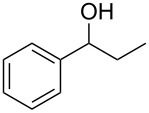	B	CH_2_Cl_2_	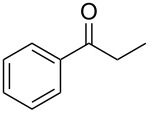	89%	85%
10	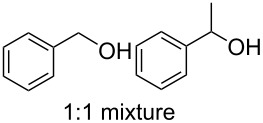	C^c^	CH_2_Cl_2_	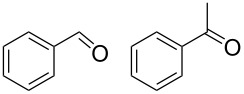	79%/16%	71%/11%
11	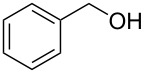	A	EtOAc	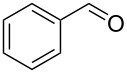	84%	81%

^a^Method A: Organic Phase – Alcohol (0.2 M) in CH_2_Cl_2_ or EtOAc set to 44 μL min^−1^ (8.8 μmol alcohol min^−1^, 1.0 equiv min^−1^). Aqueous Phase – Aqueous NaOCl (0.25 M), adjusted to pH 9.1 with NaHCO_3_, mixed with aqueous KBr (0.5 M, 30 µL per mL NaOCl) set to 56 µL min^−1^ (1.5 equiv NaOCl min^−1^, 0.10 equiv KBr min^−1^). The phases combined at a Y-junction and passed through a 60 cm channel packed with AO-TEMPO (300 mg, 0.138 mmol TEMPO) submerged in an ice bath. ^b^Method B: Organic Phase – Alcohol (0.1 M) in CH_2_Cl_2_ set to 44 μL min^−1^ (4.4 μmol alcohol min^−1^, 1.0 equiv min^−1^). Aqueous Phase – Aqueous NaOCl (0.25 M), adjusted to pH 9.1 with NaHCO_3_, mixed with aqueous KBr (0.5 M, 30 µL per mL NaOCl) set to 56 µL min^−1^ (3.0 equiv NaOCl min^−1^, 0.20 equiv KBr min^−1^). The phases combined at a Y-junction and passed through a 60 cm channel packed with AO-TEMPO (300 mg, 0.138 mmol TEMPO) submerged in an ice bath. ^c^Method C: Organic Phase – Benzyl alcohol (0.2 M) and 1-phenylethanol (0.2 M) in CH_2_Cl_2_ set to 44 μL min^−1^ (8.8 μmol alcohol min^−1^, 1.0 equiv min^−1^). Aqueous Phase – Aqueous NaOCl (0.20 M), adjusted to pH 9.1 with NaHCO_3_, mixed with aqueous KBr (0.5 M, 30 µL per mL NaOCl) set to 56 µL min^−1^ (1.25 equiv NaOCl min^−1^, 0.10 equiv KBr min^−1^). The phases combined at a Y-junction and passed through a 60 cm channel packed with AO-TEMPO (300 mg, 0.138 mmol TEMPO) submerged in an ice bath. ^d^Conversions and yields determined by GC using cyclooctane as an internal standard. ^e^Number in parentheses corresponds to isolated yield.

**Figure 3:**
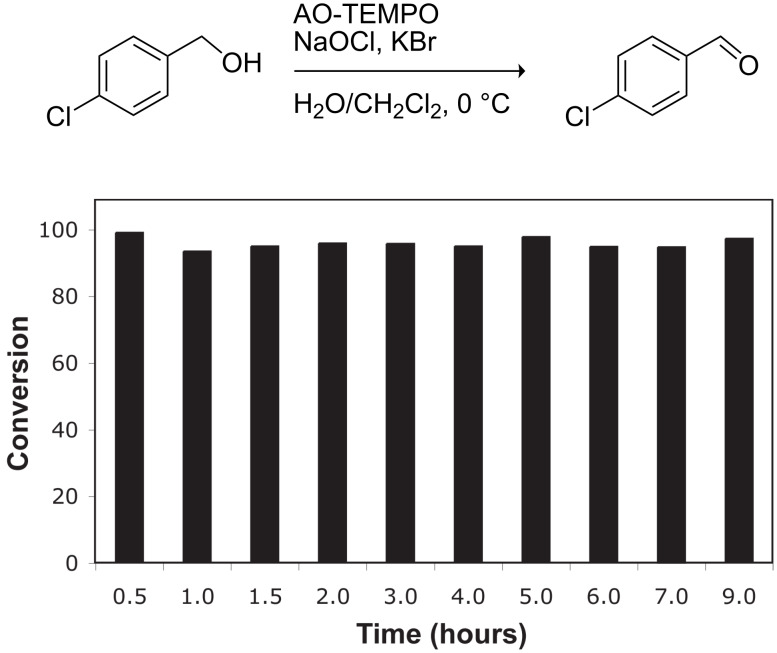
The long-term activity of AO-TEMPO packed beds in the oxidation of 4-chlorobenzyl alcohol. A solution of 4-chlorobenzyl alcohol (0.2 M in CH_2_Cl_2_) set to 44 μL min^−1^ (8.8 μmol min^−1^, 1.0 equiv min^−1^) and an aqueous solution consisting of NaOCl (0.25 M), adjusted to pH 9.1 with NaHCO_3_, and KBr (0.5 M, 30 µL per mL NaOCl) set to 56 µL min^−1^ (1.5 equiv NaOCl min^−1^, 0.10 equiv KBr min^−1^) were passed through the AO-TEMPO packed bed for 9 h. Fractions were collected and analyzed by GC using an internal standard.

To test the long-term activity of the AO-TEMPO packed beds, the oxidation of 4-chlorobenzyl alcohol to 4-chlorobenzaldehyde was run continuously and sampled periodically to monitor its activity. As seen in Figure 3, the activity of the catalyst bed remained high even after hours of use. Furthermore, the work-up of this simplified oxidation scale-up comprised only of phase separation followed by concentration, yielding a white crystalline solid with greater than 95% purity by ^1^H NMR.

## Conclusion

We have demonstrated that using supported TEMPO is an efficient method to oxidize alcohols using a simplified packed-bed microreactor. A biphasic mixture was thoroughly mixed by passing the immiscible liquids through the catalytic packed-bed, leading to no disruptions or degradation of the packing material. Thus, the AO-TEMPO resins are recyclable, showing no loss of catalytic activity and a substrate scope that encompasses many primary and secondary alcohols. The devices presented are predicted to be readily scaled-up to achieve the desired output of a reaction and is of higher throughput than other reported packed-bed microreactors.

## Experimental

### General

Solvents were purified by standard procedures. All other reagents were used as received, unless otherwise noted. Sodium hypochlorite solution (reagent grade, available chlorine 10–15%) was purchased from Aldrich and titrated before use. ^1^H NMR and ^13^C NMR spectra were recorded in CDCl_3_ on Varian Mercury 300 MHz operating at 300.070 MHz and 75.452 MHz, respectively, using the residual solvent peak as reference. ATR-IR was performed on a Nicolet Avatar DTGS 370 infrared spectrometer with Avatar OMNI sampler and OMNIC software. Elemental analysis was performed by Robertson Microlit Laboratories, Inc., in Madison, New Jersey. Gas chromatographic (GC) analyses were performed using an Agilent 7890A GC equipped with an Agilent 7683B autosampler, a flame ionization detector (FID), and a J&W Scientific 19091J-413 column (length = 30 m, inner diameter = 320 μm, and film thickness = 250 μm). The temperature program for GC analysis held the temperature constant at 80 °C for 1 min, heated samples from 80 to 200 °C at 20 °C/min and held at 200 °C for 1 min. Inlet and detector temperatures were set constant at 220 and 250 °C, respectively. Cyclooctane was used as an internal standard to calculate reaction conversion and yield. Gas chromatography-mass spectrometry (GC/MS) analyses were performed using a Hewlett Packard HP 6890 Series Gas Chromatograph, a Hewlett Packard HP 5973 Mass Spectrometer Detector (MSD), and a J&W Scientific DB*-5 Column (length = 30 m, inner diameter = 0.325 mm, film thickness = 1.0 µm, catalog number 123-5033). The temperature program for the analyses held the temperature constant at 50 °C for 3 min, heated samples from 50 to 80 °C at 30 °C/min, holding at 80 °C for 2 min, then heating samples from 80 to 200 °C at 17 °C/min, and holding at 200 °C for 1.94 min. The MSD temperature was held at 300 °C for 15 min.

### Azide modified AMBERZYME^®^ Oxirane (AO-N_3_, **2**)

Sodium azide (5.26 g, 81 mmol, 8.1 equiv) and ammonium chloride (2.27 g, 42.4 mmol, 4.2 equiv) were dissolved in 500 mL 90:10 v/v water in methanol. AMBERZYME^®^ Oxirane (10.0 g, 1.0 mmol epoxide/g resin, 10.0 mmol, 1.0 equiv) was suspended in the azide solution and the reaction mixture refluxed overnight with gentle stirring. The resin was filtered using a Buchner funnel, washed with deionized H_2_O (2 × 50 mL), MeOH (2 × 50 mL), Et_2_O (1 × 25 mL), and dried under vacuum. Elemental analysis afforded a loading of 1.0 mmol N_3_/g resin.

### Propargyl ether TEMPO **5**

Sodium hydride (150 mg, 6.3 mmol, 1.1 equiv) was added to DMF (10 mL) and stirred at RT. 4-hydroxy-TEMPO (1.02 g, 5.9 mmol, 1.0 equiv) in DMF (10 mL) was added drop wise to the sodium hydride suspension at 0 °C and stirred until gas evolution ceased. Propargyl bromide (80% in toluene, 800 μL, 7.4 mmol, 1.3 equiv) in DMF (10 mL) was added at 0 °C and the reaction was allowed to warm up to RT and stirred overnight. The reaction was quenched with water and the aqueous phase was extracted with ethyl acetate (3 × 50 mL) and the combined organic extracts dried over MgSO_4_, concentrated and dried under vacuum. The product was purified using column chromatography (silica gel, 1:1 → 1:2 hexanes/ethyl acetate, *R_f_* = 0.40) to yield a dark orange solid (841 mg, 68%). δ_C_ (75 MHz, CDCl_3_): 20.619, 32.199, 44.530, 55.298, 59.198, 69.905, 74.238; MS *m/z* 210 (M^+^). To obtain NMR spectra, a few drops of phenylhydrazine were added to the NMR tube to reduce the product to the corresponding hydroxylamine.

### AO-TEMPO **6**

**5** (450 mg, 2.14 mmol, 1.3 equiv) and CuI (40 mg, 0.2 mmol, 0.13 equiv) were dissolved in anhydrous THF (20 mL). **2** (1.64 g, 1.0 mmol N_3_/g resin, 1.64 mmol, 1.0 equiv) was added to the solution and placed under a N_2_ atmosphere. The suspension was shaken for 3 d. The resin was washed with THF (2 × 10 mL), MeOH (1 × 10 mL), 1 M HCl (2 × 10 mL), deionized H_2_O (1 × 10 mL), sat. NaHCO_3_ (2 × 10 mL), deionized H_2_O (1 × 10 mL), MeOH (1 × 10 mL) and CH_2_Cl_2_ (1 × 10 mL), and dried under vacuum to yield the white AO-TEMPO resin (1.81 g, 0.46 mmol TEMPO/g resin). The loading was calculated by mass difference.

## Supporting Information

File 1 Experimental methods and spectral data
