# Supplementary material for: A biphasic oxidation of alcohols to aldehydes and ketones using a simplified packed-bed microreactor
Source: Beilstein J Org Chem. 2009 Apr 29;5:17. doi: 10.3762/bjoc.5.17 (PMC2686313; doi:10.3762/bjoc.5.17)

A biphasic oxidation of alcohols to aldehydes and ketones using a simplified packed-bed microreactor

Andrew Bogdan[1] and D. Tyler McQuade[2,*]

Address: [1]Department of Chemistry and Chemical Biology, Cornell University, Ithaca, NY, 14853 USA and [2]Department of Chemistry and Biochemistry, Florida State University, Tallahassee, FL 32306 USA. Fax (850) 644-8281

Email: D. Tyler McQuade* - mcquade@chem.fsu.edu

* Corresponding author

Supplementary Information

General: Solvents were purified by standard procedures. All other reagents were used as received, unless otherwise noted. Sodium hypochlorite solution (reagent grade, available chlorine 10–15%) was purchased from Aldrich and titrated before use. ^{1}H NMR and ^{13}C NMR spectra were recorded in $CDCl_3$ on Varian Mercury 300 MHz operating at 300.070 MHz and 75.452 MHz, respectively, using the residual solvent peak as reference. ATR-IR was performed on a Nicolet Avatar DTGS 370 infrared spectrometer with Avatar OMNI sampler and OMNIC software. Elemental analysis was performed by Robertson Microlit Laboratories, Inc., in Madison, New Jersey. Gas chromatographic (GC) analyses were performed using an Agilent 7890A GC equipped with an Agilent 7683B autosampler, a flame ionization detector (FID), and a J&W Scientific 19091J-413 column (length = 30 m, inner diameter = 320 μm, and film thickness = 250 μm). The temperature program for GC analysis held the temperature constant at 80 ˚C for 1 min, heated samples from 80 to 200 ˚C at 20 ˚C/min and held at 200 ˚C for 1 min. Inlet and detector temperatures were set constant at 220 and 250 ˚C, respectively. Cyclooctane was used as an internal standard to calculate reaction conversion and yield. Gas chromatography-mass spectrometry (GC/MS) analyses were performed using a Hewlett Packard HP 6890 Series Gas Chromatograph, a Hewlett Packard HP 5973 Mass Spectrometer Detector (MSD), and a J&W Scientific DB*-5 Column (length = 30 m, inner diameter = 0.325 mm, film thickness = 1.0 μm, catalog number 123-5033). The temperature program for the analyses held the temperature constant at 50 ˚C for 3 min, heated samples from 50 to 80 ˚C at 30 ˚C/min, holding at 80 ˚C for 2 min, then

heating samples from 80 to 200 ˚C at 17 ˚C/min, and holding at 200 ˚C for 1.94 min. The MSD temperature was held at 300 ˚C for 15 min.

Channel packing: A 60 cm section of tubing (Swagelok PFA Tubing, 1/8" outer diameter) was attached to a Swagelok PFA union packed with glass wool. A separate union (not packed with glass wool) was attached to the inlet side of the 60 cm column. Two 10 cm sections of tubing were attached to the channel, serving as the outlet. A 1/16" female Luer was attached to the inlet. Functionalized resin was suspended in CH_2Cl_2 and the column slurry packed using a syringe.

Residence time determination: Three columns (10 cm, 30 cm, and 60 cm) were packed with AO resin. A dye solution was prepared by dissolving 15 mg of Disperse Red 1 in 20 mL CH_2Cl_2. A 5 mL syringe was filled with the dye and passed through the channel. Using a flow rate of 2.0 mL/min, the residence time was determined for all three packed columns. The residence time was measured from initial appearance of the dye on the column to the appearance of a pink hue at the outlet of the channel. This was repeated in triplicate for all three column lengths and residence time increased linearly with channel length. Between trials, the channel was rinsed with 3 mL of CH_2Cl_2.

Column length (cm)	Average residence time (s)	St. dev.
60	13.82	0.33
30	5.90	0.35
10	1.75	0.13

A second set of experiments was conducted to determine the relationship between the flow rate and residence time. A 30 cm column was packed with 137 mg of AO. The residence times were measured using the above procedure.

Flow rate (mL/min)	Average residence time (s)	St. dev.
3.90	2.60	0.05
2.00	5.05	0.45
1.00	11.20	0.68
0.40	30.56	0.99
0.20	68.20	1.73
0.10	141.70	2.83

Aqueous flow rate (μL min^{-1})	Organic flow rate (μL min^{-1})	Approx. residence time (s)
224	176	62
112	88	134
56	44	288

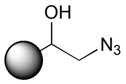

Azide modified AMBERZYME® Oxirane (AO-N_3, 2): Sodium azide (5.26 g, 81 mmol, 8.1 equiv) and ammonium chloride (2.27 g, 42.4 mmol, 4.2 equiv) were dissolved in 500 mL 90:10 v/v water in methanol. AMBERZYME® Oxirane (10.0 g, 1.0 mmol epoxide/g resin, 10.0 mmol, 1.0 equiv) was suspended in the azide solution and the reaction mixture refluxed overnight with gentle stirring. The resin was filtered using a Buchner funnel, washed with deionized H_2O (2x 50 mL), MeOH (2x 50 mL), Et_2O (1x 25 mL), and dried under vacuum. Elemental analysis afforded a loading of 1.0 mmol N_3/g resin.

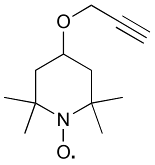

Propargyl ether TEMPO 5: Sodium hydride (150 mg, 6.3 mmol, 1.1 equiv) was added to DMF (10 mL) and stirred at RT. 4-hydroxy-TEMPO (1.02 g, 5.9 mmol, 1.0 equiv) in DMF (10 mL) was added drop wise to the sodium hydride suspension at 0 ˚C and stirred until gas evolution ceased. Propargyl bromide (80% in toluene, 800 μL, 7.4 mmol, 1.3 equiv) in DMF (10 mL) was added at 0 ˚C and the reaction was allowed to warm up to RT and stirred overnight. The reaction was quenched with water and the aqueous phase was extracted with ethyl acetate (3x 50 mL) and the combined organic extracts dried over $MgSO_4$, concentrated and dried under vacuum. The product was purified using column chromatography (silica gel, 1:1 → 1:2 hexanes/ethyl acetate, R_f = 0.40) to yield a dark orange solid (841 mg, 68%). δ_C (75 MHz, $CDCl_3$): 20.619, 32.199, 44.530, 55.298, 59.198, 69.905, 74.238; MS *m/z* 210 (M^+). To obtain NMR spectra, a few drops of phenylhydrazine were added to the NMR tube to reduce the product to the corresponding hydroxylamine.

AT-IR compound 5

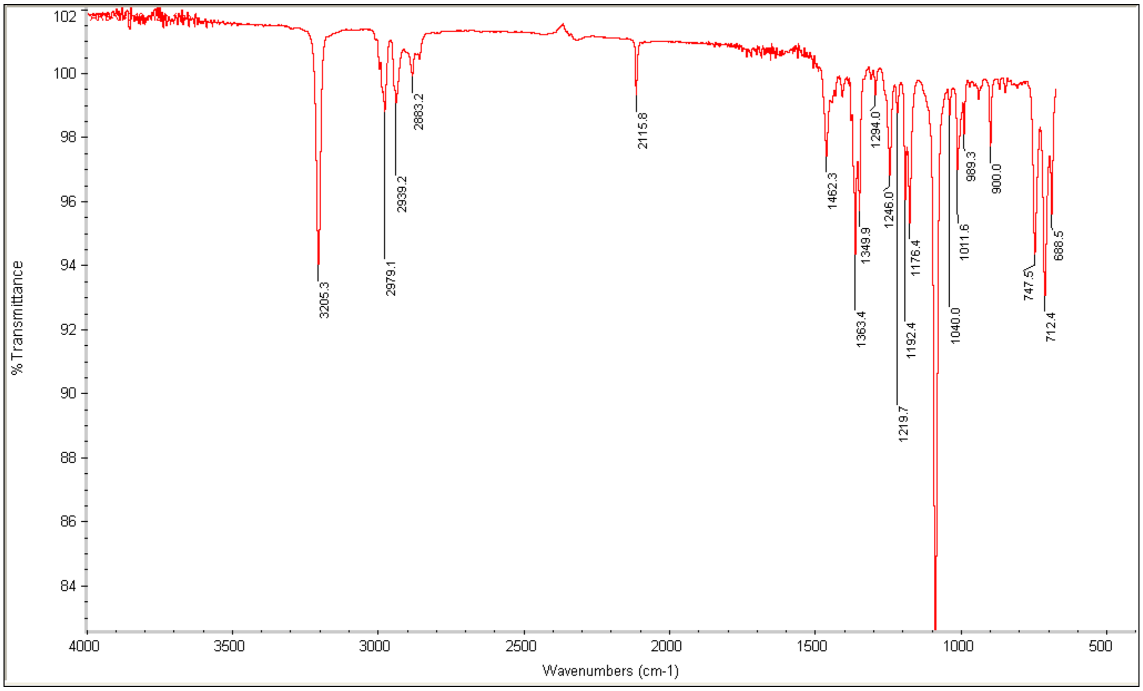

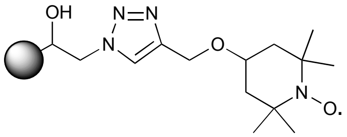

AO-TEMPO 6: 5 (450 mg, 2.14 mmol, 1.3 equiv) and CuI (40 mg, 0.2 mmol, 0.13 equiv) were dissolved in anhydrous THF (20 mL). **2** (1.64 g, 1.0 mmol N_3/g resin, 1.64 mmol, 1.0 equiv) was added to the solution and placed under a N_2 atmosphere. The suspension was shaken for 3 d. The resin was washed with THF (2x 10 mL), MeOH (1x 10 mL), 1 M HCl (2x 10 mL), deionized H_2O (1x 10 mL), sat. $NaHCO_3$ (2x 10 mL), deionized H_2O (1x 10 mL), MeOH (1x 10 mL) and CH_2Cl_2 (1x 10 mL), and dried under vacuum to yield the white AO-TEMPO resin (1.81 g, 0.46 mmol TEMPO/g resin). The loading was calculated by mass difference.

Representative primary alcohol flow oxidation procedure: Benzyl alcohol (0.2 M) and cyclooctane (0.02 M) in CH_2Cl_2 or EtOAc was placed in a syringe and placed on a syringe pump set to 44 μL min^{-1} (8.8 μmol alcohol min^{-1}, 1.0 equiv min^{-1}). Aqueous NaOCl (purchased from Sigma Aldrich and diluted to 0.25 M), adjusted to pH 9.1 with $NaHCO_3$, mixed with aqueous KBr (0.5 M, 30 μL per mL NaOCl) were placed in a separate syringe and placed on another syringe pump set to 56 μL min^{-1} (1.5 equiv NaOCl min^{-1}, 0.10 equiv KBr min^{-1}). The phases combined at a Y-junction and passed through a 60 cm channel packed with AO-TEMPO (300 mg) submerged in an ice bath. The reaction mixture was collected in a 4 mL screw cap vial until the sample had eluted through the channel. Upon completion of each experiment, the organic phase was separated from the aqueous phase using a pipette and transferred to a separate 4 mL screw cap vial. After each trial, the channel was washed with 5 mL deionized H_2O, 5 mL MeOH, and 5 mL CH_2Cl_2. The column was flushed with air prior to each run. Samples were analyzed by GC and conversions and yields obtained by comparing starting material and product to an internal standard.

Representative secondary alcohol flow oxidation procedure: 1-phenylethanol (0.1 M) and cyclooctane (0.01 M) in CH_2Cl_2 was placed in a syringe and placed on a syringe pump set to 44 μL min^{-1} (4.4 μmol alcohol min^{-1}, 1.0 equiv min^{-1}). Aqueous NaOCl (purchased from Sigma Aldrich and diluted to 0.25 M), adjusted to pH 9.1 with $NaHCO_3$, mixed with aqueous KBr (0.5 M, 30 μL per mL NaOCl) were placed in a separate syringe and placed on another syringe

pump set to 56 μL min^{-1} (3.0 equiv NaOCl min^{-1}, 0.20 equiv KBr min^{-1}). The phases combined at a Y-junction and passed through a 60 cm channel packed with AO-TEMPO (300 mg) submerged in an ice bath. The reaction mixture was collected in a 4 mL screw cap vial until the sample had eluted through the channel. Upon completion of each experiment, the organic phase was separated from the aqueous phase using a pipette and transferred to a separate 4 mL screw cap vial. After each trial, the channel was washed with 5 mL deionized H_2O, 5 mL MeOH, and 5 mL CH_2Cl_2. The column was flushed with air prior to each run. Samples were analyzed by GC and conversions and yields obtained by comparing starting material and product to an internal standard.

Bleach titration: Sodium hypochlorite solution (Sigma Aldrich, reagent grade, 500 μL) was diluted to 100 mL with deionized H_2O in an Erlenmeyer flask equipped with a stir bar. Excess KI and excess H_2SO_4 (conc.) were added to the flask, yielding a dark burgundy solution. Sodium thiosulfate pentahydrate (1.0 M in deionized H_2O) was added drop wise to the flask until the color of the solution dissipated (the equivalence point). The concentration of NaOCl in the solution was calculated using the following reactions.

$$ClO^- + 2\,H^+ + 2\,I^- \rightleftharpoons Cl^- + I_2 + H_2O$$

$$I_2 + 2\,S_2O_3^{2-} \rightleftharpoons 2\,I^- + S_4O_6^{2-}$$

$$M\ NaOCl = \frac{0.5 \times mmol\ Na_2S_2O_3}{0.5\ mL\ NaOCl\ solution}$$

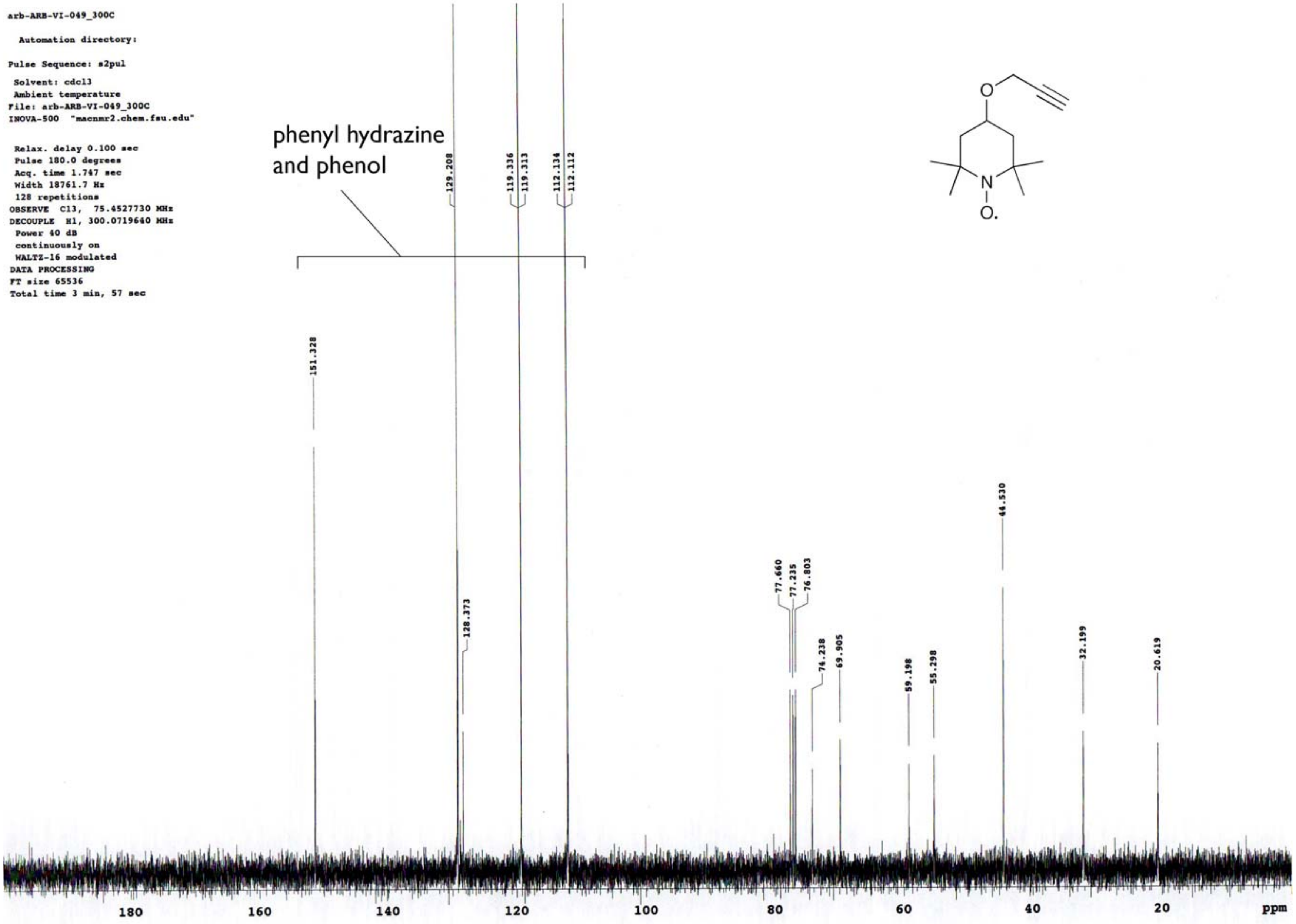
arb-ARB-VI-049_300C
Automation directory:
Pulse Sequence: s2pul
Solvent: cdcl3
Ambient temperature
File: arb-ARB-VI-049_300C
INOVA-500 "macnmr2.chem.fsu.edu"
Relax. delay 0.100 sec
Pulse 180.0 degrees
Acq. time 1.747 sec
Width 18761.7 Hz
128 repetitions
OBSERVE C13, 75.4527730 MHz
DECOUPLE H1, 300.0719640 MHz
Power 40 dB
continuously on
WALTZ-16 modulated
DATA PROCESSING
FT size 65536
Total time 3 min, 57 sec
phenyl hydrazine
and phenol
151.328
129.208
128.373
119.336
119.313
112.134
112.112
77.660
77.235
76.803
74.238
69.905
59.198
55.298
44.530
32.199
20.619
180
160
140
120
100
80
60
40
20
ppm

ARB-VI-212_300H

Automation directory:

Pulse Sequence: s2pul

Solvent: cdcl3
Ambient temperature
File: arb-ARB-VI-212_300H
INOVA-500 "macnmr2.chem.fsu.edu"

Relax. delay 1.000 sec
Pulse 22.5 degrees
Acq. time 1.666 sec
Width 4909.2 Hz
16 repetitions
OBSERVE H1, 300.0704275 MHz
DATA PROCESSING
FT size 16384
Total time 0 min, 42 sec

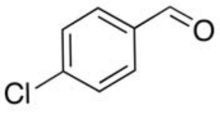

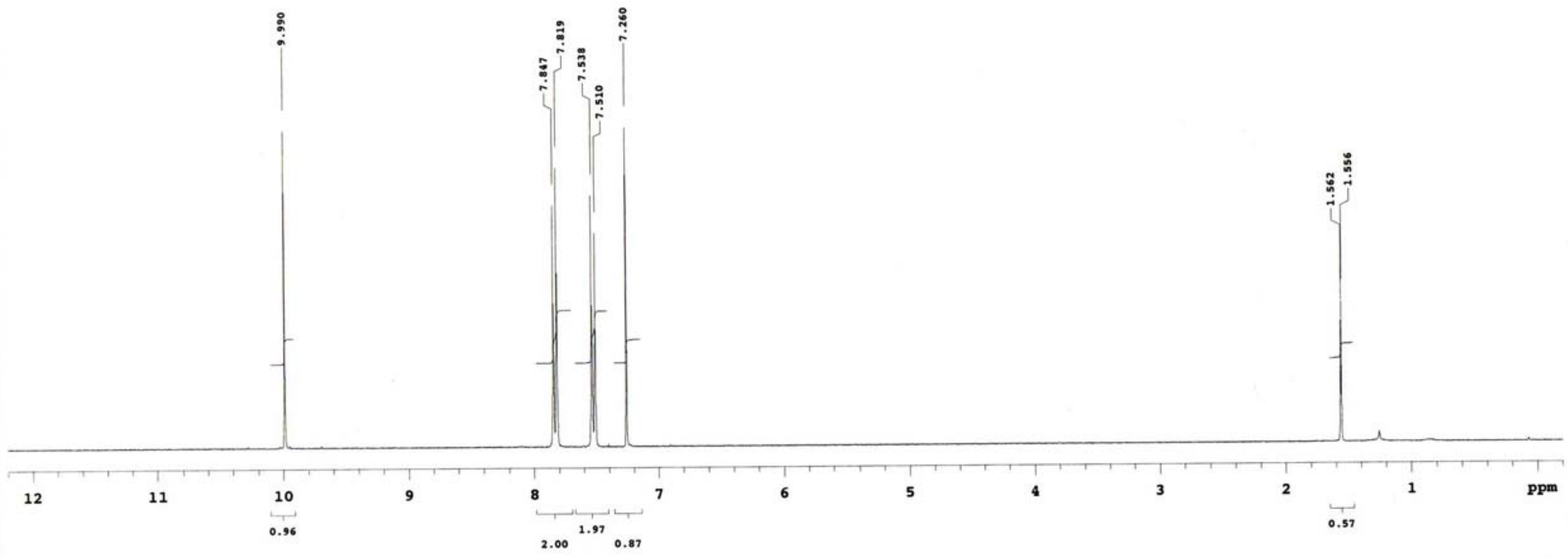

Supplement: File 1 — Experimental methods and spectral data [file Beilstein_J_Org_Chem-05-17-s001.pdf]
